# Inhibitory Network Bistability Explains Increased Interneuronal Activity Prior to Seizure Onset

**DOI:** 10.3389/fncir.2019.00081

**Published:** 2020-01-14

**Authors:** Scott Rich, Homeira Moradi Chameh, Marjan Rafiee, Katie Ferguson, Frances K. Skinner, Taufik A. Valiante

**Affiliations:** ^1^Division of Clinical and Computational Neuroscience, Krembil Research Institute, University Health Network, Toronto, ON, Canada; ^2^Departments of Medicine (Neurology) and Physiology, University of Toronto, Toronto, ON, Canada; ^3^Institute of Biomaterials and Biomedical Engineering, University of Toronto, Toronto, ON, Canada; ^4^Institute of Medical Science, University of Toronto, Toronto, ON, Canada; ^5^Division of Neurosurgery, Department of Surgery, University of Toronto, Toronto, ON, Canada; ^6^Department of Electrical and Computer Engineering, University of Toronto, Toronto, ON, Canada

**Keywords:** epilepsy, seizure, bistability, computational neuroscience, synchrony, inhibitory network, interneurons

## Abstract

Recent experimental literature has revealed that GABAergic interneurons exhibit increased activity prior to seizure onset, alongside additional evidence that such activity is synchronous and may arise abruptly. These findings have led some to hypothesize that this interneuronal activity may serve a causal role in driving the sudden change in brain activity that heralds seizure onset. However, the mechanisms predisposing an inhibitory network toward increased activity, specifically prior to ictogenesis, without a permanent change to inputs to the system remain unknown. We address this question by comparing simulated inhibitory networks containing control interneurons and networks containing hyperexcitable interneurons modeled to mimic treatment with 4-Aminopyridine (4-AP), an agent commonly used to model seizures *in vivo* and *in vitro*. Our *in silico* study demonstrates that model inhibitory networks with 4-AP interneurons are more prone than their control counterparts to exist in a bistable state in which asynchronously firing networks can abruptly transition into synchrony driven by a brief perturbation. This transition into synchrony brings about a corresponding increase in overall firing rate. We further show that perturbations driving this transition could arise *in vivo* from background excitatory synaptic activity in the cortex. Thus, we propose that bistability explains the increase in interneuron activity observed experimentally prior to seizure via a transition from incoherent to coherent dynamics. Moreover, bistability explains why inhibitory networks containing hyperexcitable interneurons are more vulnerable to this change in dynamics, and how such networks can undergo a transition without a permanent change in the drive. We note that while our comparisons are between networks of control and ictogenic neurons, the conclusions drawn specifically relate to the unusual dynamics that arise prior to seizure, and not seizure onset itself. However, providing a mechanistic explanation for this phenomenon specifically in a pro-ictogenic setting generates experimentally testable hypotheses regarding the role of inhibitory neurons in pre-ictal neural dynamics, and motivates further computational research into mechanisms underlying a newly hypothesized multi-step pathway to seizure initiated by inhibition.

## 1. Introduction

**Figure 1:**
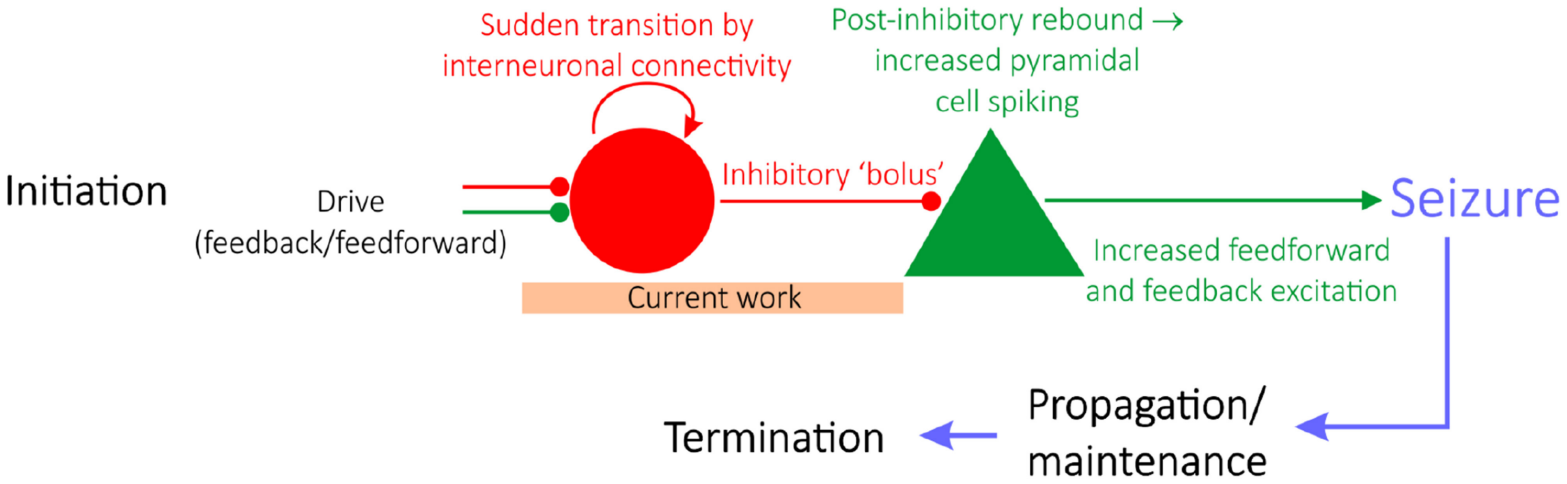
A “GABAergic initiation hypothesis” in the context of the state of epilepsy research. Epilepsy research is divided into studies focusing on seizure initiation (e.g., Miri et al., 2018), propagation (e.g., Trevelyan et al., 2006; Ellender et al., 2014), or termination (e.g., Schindler et al., 2007). Given the focus on interneuronal dynamics prior to seizure, this work sheds light on a “GABAergic initiation hypothesis” of seizure, diagrammed in detail here. The articulation of a potential mechanism explaining the sudden transition of interneurons into synchrony, alongside a justification as to why networks in a seizure state are more vulnerable to this transition, should be identified in order for this overall hypothesis of seizure initiation to be viable.

Epilepsy is a neurological condition distinguished by repeated seizures, characterized by seemingly synchronous activity of pyramidal neurons. Epilepsy research is typically divided into studies focused on either seizure initiation (Miri et al., 2018), propagation (Trevelyan et al., 2006; Ellender et al., 2014), or termination (Schindler et al., 2007), as schematized in Figure 1 (Jiruska et al., 2013). Historically, studies of seizure initiation have focused on the hypothesis that hyperexcitability of excitatory cells is the impetus for seizures (Jiruska et al., 2013) with an associated inhibitory collapse.

More recently, studies of seizure initiation have shifted focus to the over-activity of inhibitory interneurons. This literature reveals that interneurons are hyperactive prior to seizure onset (Lillis et al., 2012; Muldoon et al., 2015; Elahian et al., 2018), alongside convincing evidence that interneurons might serve a causal role in seizure initiation (Klaassen et al., 2006; Avoli and de Curtis, 2011; Avoli et al., 2016; Librizzi et al., 2017; Chang M. et al., 2018; Elahian et al., 2018; Miri et al., 2018). Such insights underlie a novel hypothesis of seizure initiation (a “GABAergic initiation hypothesis”) in which synchronous activation of inhibitory interneurons can precipitate the onset of a seizure, as diagrammed in Figure 1 (Chang M. et al., 2018). Given the contemporary nature of this hypothesis and increased pre-ictal interneuronal activity, the unique dynamics exhibited by interneurons prior to seizure are an ideal target for rigorous computational study. Here, such research aims to propose a viable process explaining the predisposition of inhibitory interneurons in a pro-ictogenic environment to suddenly increase their firing as seen experimentally, potentially alongside synchronous firing, as the system moves toward seizure. We thus focus on modeling dynamics prior to seizure initiation, at most including the earliest time in the transition to seizure.

Computational studies are uniquely suited to articulate mechanisms using the language of neural dynamics. While no *in silico* model contains the complexity of the biology, that complexity also makes uncovering mechanisms of action especially difficult *in vivo* and *in vitro*; indeed, the interactions of various facets of the brain dictating overall behavior are complex, non-linear, and multi-scale. Mathematical models provide a powerful tool for disentangling such biological complexity and proposing hypotheses. Such models inherently require abstracting the biology of the system. However, when these approximations are fully rationalized, and the conclusions appropriately constrained by the underlying assumptions in the *in silico* model, the findings from such studies can be translated into experimentally testable hypotheses (with “*a priori*” support arising mathematically or computationally) guiding future *in vitro* or *in vivo* studies.

Networks of purely inhibitory neurons have been of great interest to computational neuroscientists given their amenability to study with computational and mathematical techniques. Networks dominated by the activity and reciprocal interactions of inhibitory interneurons exist biologically, playing a key role in the generation of gamma rhythms (Whittington et al., 1995; Bartos et al., 2007; Sohal et al., 2009) and sharp-wave ripples (Schlingloff et al., 2014). However, experiments alone are often insufficient to provide us mechanistic explanations of these neural dynamics. Meanwhile, *in silico* studies are uniquely situated to discern such mechanisms by making reasonable approximations of these biological networks into modeled networks. Oftentimes, this is done by “averaging” the activity of excitatory pyramidal neurons into some external input to the system, which allows for the direct probing of how properties of the interneurons and their connectivity affect network dynamics. It is worth noting that, for this study, our interest in epilepsy does not undermine the reasonableness of this approximation, considering that we are interested only in the system prior to seizure onset. In this setting, when no seizure-like activity in pyramidal cells has yet arisen, it is reasonable to approximate the physiological activity of these neurons into a tonic external drive to the inhibitory network.

Of particular interest in the computational study of purely inhibitory networks is their tendency to synchronize, which dates back to the work of Wang and Rinzel (1992). Various mechanisms have been proposed to explain the generation of oscillations in purely inhibitory networks, the most prominent of which may be the Interneuron Network Gamma (ING) mechanism (Traub et al., 1998; Whittington et al., 2000; Bartos et al., 2007; Tiesinga and Sejnowski, 2009; Wang, 2010; ter Wal and Tiesinga, 2015). Previous work has shown that inhibitory networks built to examine population activity in an *in vitro* hippocampal preparation manifest “sharp transitions” into coherent population activity caused by a small, permanent increase to the external drive to the network (Ferguson et al., 2013). Additional studies have explored the effect of connection probabilities and cell characteristics manifested by classifications of cell excitability on inhibitory network synchrony (Tikidji-Hamburyan et al., 2015; Rich et al., 2016), and have noted that bistability between asynchronous and synchronous firing was possible (Rich et al., 2016). More recently, Tikidji-Hamburyan et al. (2019) have examined in great detail the stability of “clustered” solutions in these types of networks, noting a specific link to the biology in both the mechanism underlying the dynamic of interest (the phase response curve, or PRC) and the application of the transitions between these states (which may relate to changes in cognitive states). Further examples of how the study of this synchrony has direct application to the brain are found in the study of the onset of sharp wave ripples in the hippocampus (Schlingloff et al., 2014; Gulyás and Freund, 2015). Indeed, the computational literature studying purely inhibitory networks is rich and has provided important insights into the roles interneurons play in experimentally observed neural dynamics.

The existing computational insights into inhibitory network dynamics, combined with the experimental literature describing the hyperactive, and potentially synchronous, activity of such networks prior to seizure, motivate this computational study. To explore why these dynamics might arise particularly in pro-ictogenic settings, randomly connected, purely inhibitory network models are developed utilizing cell models mimicking properties exhibited by neurons treated with 4-Aminopyridine (4-AP), a commonly used experimental model to generate seizures (Perreault and Avoli, 1991; Williams and Hablitz, 2015), or properties of a healthy, control interneuron. Utilizing these tools, this investigation articulates a mechanism underlying a sudden transition from asynchronous to synchronous firing in an inhibitory network through which an increase in interneuronal firing rates might arise. Crucially, this mechanism also offers an explanation for the predisposition of hyperexcitable networks toward this transition.

It is worth noting that, while there are a multitude of biological effects of 4-AP beyond the hyperexcitability induced by potassium channel blockade, we focus here on the hyperexcitability it induces given our desire to explain our previous observations (Chang M. et al., 2018) as well as the plethora of contemporary studies that use the 4-AP seizure model to understand seizure mechanisms (Perreault and Avoli, 1991; Kibler and Durand, 2011; Williams and Hablitz, 2015; Baird-Daniel et al., 2017; Wenzel et al., 2017; Chang M. et al., 2018; Liou et al., 2018; Chang et al., 2019; Shivacharan et al., 2019). This feature is considered the driving force of the pro-ictogenic properties of this acute seizure model (Chang et al., 2019; Shivacharan et al., 2019); indeed, recent work has indicated that 4-AP induces seizures independent of its effects on synapses (Shivacharan et al., 2019). A specific advantage of 4-AP, noted recently by Baird-Daniel et al. (2017), is that it preserves inhibitory mechanisms, making it especially useful in the study of interneuronal dynamics at seizure initiation.

Using computational modeling, we uncovered a “bistable transition” mechanism that drives an inhibitory network into synchrony by comparing the tendency of control and 4-AP inhibitory networks to synchronize. We found that under 4-AP conditions, networks are much more likely to transition from asynchronous to synchronous dynamics following a perturbation due to a notably larger regime of network parameters supporting bistability. Additionally, such synchrony was accompanied by an increased firing rate of neurons similar to what is seen *in vivo*. The existence of such a bistable transition driving an inhibitory network into synchrony expands upon existing literature probing such mechanisms, especially in the context of epilepsy. These findings provide a *in silico* mechanistic explanation for the inhibitory dynamics observed during the transition to seizure, providing support for the potential complicity of inhibitory interneurons in seizure initiation (Klaassen et al., 2006; Avoli and de Curtis, 2011; Lillis et al., 2012; Muldoon et al., 2015; Avoli et al., 2016; Librizzi et al., 2017; Chang M. et al., 2018; Elahian et al., 2018; Miri et al., 2018).

## 2. Materials and Methods

Using optogenetic mice expressing channelrhodopsin-2 in inhibitory interneurons under proconvulsant conditions of 4-AP (Voskuyl and Albus, 1985), it has been shown that the activation of inhibitory interneurons in layer 2–3 (L2/3) of mouse somatosensory cortex can trigger ictal events (Chang M. et al., 2018). The strategy here involved building generic inhibitory networks that roughly approximate cortical inhibitory networks, utilizing neuron models of both a healthy, control interneuron and an interneuron made hyperexcitable by treatment with 4-AP. Such an undertaking was informed by a combination of existing computational models of inhibitory interneurons, literature describing the general effects of 4-AP, and unpublished in-house experiments yielding data from the same interneuron in both control and 4-AP settings.

### 2.1. Neuron Models

Neurons were modeled via a two dimensional system of ordinary differential equations first described by Izhikevich (Izhikevich, 2003). This model has two variables: *V*, which represents the membrane potential in mV; and *u*, which represents the slow “recovery” current in pA. The model utilized here is slightly altered in the fashion described by Ferguson et al. (2013), and is given by:

(1) $$\begin{array}{l} \;C_{m}\;\dot{V}=k(V-v_{r})(V-v_{t})-u-I_{syn}+I_{app}+I_{perturb}\\ \;\dot{u}=a[b(V-v_{r})-u]\\ \text{ if }V\geq v_{peak},\text{then }V\leftarrow c\text{ and }u\leftarrow u+d\\ \text{where }k=k_{low}\text{ if }V\leq v_{t}\text{ and }k=k_{high}\text{ if }V>v_{t} \end{array}$$

In the above equations, *C*_*m*_ represents the membrane capacitance in pF, *v*_*r*_ represents the resting membrane potential in mV, *v*_*t*_ represents the instantaneous threshold potential in mV, *v*_*peak*_ is the spike cut-off value in mV, *I*_*syn*_ is sum of all incoming synaptic current to the neuron in pA (described in detail below), *I*_*app*_ represents the external applied current in pA (described in detail below), *I*_*perturb*_ represents the perturbation current in pA (described in detail below), *a* is the recovery time constant of the adaptation current in ms^−1^, *b* describes the sensitivity of the adaptation current to subthreshold fluctuations in nS, *c* is the voltage reset value in mV, *d* is the total current affecting the after spike behavior in pA, and *k*_*low*_ and *k*_*high*_ are scaling factors in nS/mV.

The use of Izhikevich model neurons was motivated by the goals of this study: namely, here we do not strictly constrain our neuron model with experimental results, but rather create a model that more “generally” matches the properties of an interneuron in both control and 4-AP cortical settings and highlights the key differences between them (particularly those caused by hyperexcitability in 4-AP interneurons). This choice allows for the detailed investigation of the mechanisms underlying the transition into synchrony in these networks performed here.

#### 2.1.1. Neuron Model Parameters

Models and parameter values were based primarily on previous Izhikevich inhibitory cell models (Ferguson et al., 2013) and the literature describing the effects of 4-AP (Williams and Hablitz, 2015). Unpublished in-house experiments were used to supplement this literature and inform the modeling in areas in which this literature was not as detailed. These experiments highlighted specific differences in control and 4-AP settings, particularly with regards to the rheobase and capacitance.

The model presented by Ferguson et al. (2013) was used as a “starting point” for the models presented here, as the neurons of interest in that study exhibit similar major properties to the types of neurons of interest in this research. This choice informed the values of *v*_*r*_, *v*_*t*_, *c*, and *v*_*peak*_. The unpublished experimental work yielded *C*_*m*_ values for cortical interneurons. We note that the calculation of *C*_*m*_ was done identically in the control and 4-AP settings for consistency (assuming isopotentiality of the cell) in order to yield a direct comparison; however, it is likely that the differing *C*_*m*_ values measured in this fashion were influenced by the application of 4-AP making the cell more electrotonically compact. The measurement of the capacitance without the isopotentiality assumption are more subtle and involved than what was necessary for this research (Rall, 1962; Johnston and Wu, 1994). As such, we emphasize that the *C*_*m*_ values presented here are not intended to be experimentally rigorous measurements of the cell's capacitance in control and 4-AP settings, but rather “approximations” that are informative for constraining our Izhikevich model neurons and matching the experimentally observed excitability profiles.

The rest of the parameter values (*a, b, d, k*_*low*_, *k*_*high*_) were chosen through a parameter exploration to match the difference in rheobase caused by treatment of 4-AP. Unpublished in-house experiments were used for the rheobase values of control and 4-AP interneurons, as recorded in the same cell, given that such details are not available in the existing literature. An increase in spike-frequency adaptation in the 4-AP setting is also implied by the literature (Williams and Hablitz, 2015) and correspondingly influenced the determination of these parameters. As the model of Ferguson et al. (2013) was used as a reasonable model of a fast-firing inhibitory cell, the slope of the frequency-current (FI) of that neuron was used for the control case. Except for the changes caused by a shifted rheobase and the presence of adaptation, this slope was kept approximately the same for the 4-AP model. With the different rheobases, this means that the firing frequency is larger in the 4-AP model relative to control for a given input current.

**Figure 2:**
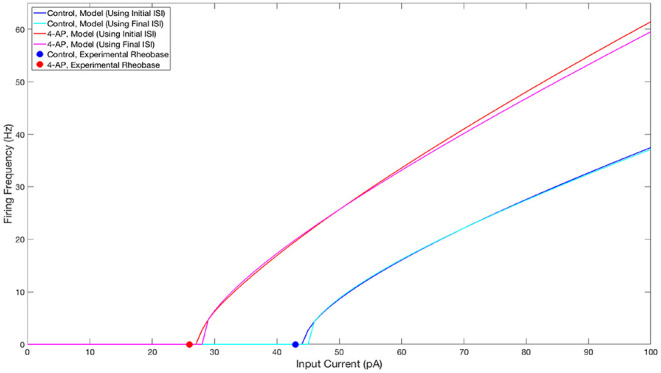
FI curves illustrating properties of neuron models used in this study. FI Curves for control (blue and cyan) and 4-AP (red and magenta) modeled neurons. Curves are shown for frequencies calculated using the initial (blue and red) and final (cyan and magenta) inter-spike intervals to illustrate the tendency for spike-frequency adaptation (SFA). These comparisons show that the neuron models utilized in this study match the decreased rheobase and increased excitability and SFA of 4-AP treated neurons in comparison to control neurons (with the rheobases determined from unpublished in-house experiment for control and 4-AP neurons highlighted on the figure by the colored dots).

The parameter values for both what will hereafter be referred to as the “control” model and what will hereafter be referred to as the “4-AP” model are included in Table 1, alongside the primary motivating factors in the choice of said parameter. Properties of these model neurons encapsulated in their FI curves are illustrated in Figure 2. All modeled neurons referred to as “control” or “4-AP” in this work use these parameter values (i.e., every neuron within a given network is identical with the exception of its external driving current).

**Table 1:** Parameters used in neuron models.

**Parameter**	**Value (Control)**	**Value (4-AP)**	**Rationale**
*C_*m*_*	73 pF	49 pF	Unpublished in-house experiment
*v_*r*_*	−60.6 mV	−60.6 mV	Ferguson et al. (2013)
*v_*t*_*	−43.1 mV	−43.1 mV	Ferguson et al. (2013)
*v_*peak*_*	2.5 mV	2.5 mV	Ferguson et al. (2013)
*a*	0.01 ms^−1^	0.01 ms^−1^	Parameter influences rheobase and adaptation exhibited by model^*^
*b*	−0.2 nS	−0.4 nS	Parameter influences rheobase and adaptation exhibited by model^*^
*c*	−67 mV	−67 mV	Ferguson et al. (2013)
*d*	0.75 pA	1.25 pA	Parameter influences rheobase and adaptation exhibited by model^*^
*k_*low*_*	0.6 nS/mV	0.4 nS/mV	Parameter influences rheobase and adaptation exhibited by model^*^
*k_*high*_*	2 nS/mV	2 nS/mV	Parameter influences rheobase and adaptation exhibited by model^*^

* *Differences in rheobase and adaptation in control and 4-AP neurons are features shown by Williams and Hablitz (2015) as well as observed in our un-published in-house experiment*.

We emphasize here that the parameters varied between the control and 4-AP model (*C*_*m*_, *b, d, k*_*low*_) act in concert to cause the differences between the 4-AP model compared to the control model. The parameter choices were not uncovered by “perturbing” individual parameters in order to elicit hyperexcitability, but rather via an investigation of the entire parameter space that uncovered sets of parameters yielding the desired dynamical properties. Our investigations did reveal that the *b* and *k*_*low*_ parameters are primarily responsible for the shifted rheobase observed in the 4-AP model compared to the control model. Similarly, the *a* and *d* values are primarily responsible for the relative amount of spike frequency adaptation exhibited by the two models.

### 2.2. Network Structure

Similar to inhibitory network models developed by Ferguson et al. (2013), the neurons in the networks modeled here were randomly connected by synapses utilizing a first-order kinetic model. Each synapse is modeled by

(2) $$\begin{array}{r} I_{syn}=g_{syn}s\left(V-E_{syn}\right) \end{array}$$

where *g*_*syn*_ is the inhibitory synaptic weight in nS, *s* is the gating variable, *V* is the membrane potential of the post-synaptic cell in mV, and *E*_*syn*_ is the inhibitory reversal potential in mV. As this value of *E*_*syn*_ is set at an inhibitory value of −75 mV for every possible synapse, this study includes *only* inhibitory synaptic connections. Furthermore, *g*_*syn*_ is uniform for each network studied, meaning each connection in a given network has the same strength.

The gating variable models the proportion of open synaptic channels, with its dynamics given by

(3) $$\begin{array}{r} \dot{s}=\alpha \left[T\right]\left(1-s\right)-\beta s \end{array}$$

where α represents the inverse of the rise time constant and β represents the inverse of the decay time constant (Destexhe et al., 1998). [*T*] models the concentration of neurotransmitter released following a pre-synaptic action potential. [*T*] is represented as a unitary pulse lasting 1 ms, from the time of the pre-synaptic spike (*t*_0_) to the end of the pulse (*t*_1_). With this, the dynamics of *s* can be simplified to the following two equations,

(4) $$\begin{array}{r} s\left(t-t_{0}\right)=s_{\infty }+\left(s\left(t_{0}\right)-s_{\infty }\right)e^{\frac{t-t_{0}}{\tau_{s}}}t_{0}<t<t_{1} \end{array}$$

(5) $$\begin{array}{r} s\left(t\right)=s\left(t_{1}\right)e^{-\beta \left(t-t_{1}\right)}t>t_{1} \end{array}$$

where $s_{\infty }=\frac{\alpha }{\alpha +\beta }$ and $\tau_{s}=\frac{1}{\alpha +\beta }$.

#### 2.2.1. Network Model Parameters

A fast rise time rate constant of α = 3.7037 ms^−1^ is used here as in Ferguson et al. (2013). Values for the inhibitory reversal potential (−75 mV) and the synaptic decay rate constant (β = 0.3333 ms^−1^) were taken from Traub et al. (2005), and the range of inhibitory synaptic conductances explored (0–10 nS) encompasses cortical estimates (Traub et al., 2005).

Network size and connectivity were based on estimates regarding the density of inhibitory cells present in the cortex and their intra-connectivity (Markram et al., 2015). Choices regarding network size were motivated by the size of L2/3 slices obtained in-house (~0.03 mm^3^) combined with observations regarding the number of large basket cells (the most abundant type of inhibitory cell in L2/3) per unit volume presented in Markram et al. (2015). Given that a single large basket cell synapses onto ~23 other large basket cells in this brain region (Markram et al., 2015), and assuming random connectivity, the probability of connection between such cells would be at least 0.04. Considering networks with connectivity densities lower than 0.04 would be unlikely to exhibit coherent dynamics in a randomly connected inhibitory network (Börgers and Kopell, 2003; Rich et al., 2016), this density is used as a lower bound for this investigation. Based on such estimations, this study utilized networks of 500 neurons with the neurons randomly connected with connection probabilities of 0.04, 0.08, 0.12, and 0.16. Connection probabilities much larger than this were not needed as they would be clearly unrealistic relative to the biological estimations.

As done in Ferguson et al. (2013), cell heterogeneity in the networks was implemented by varying the amplitude of the tonic external input current, *I*_*app*_, to each neuron. The input currents were selected from a normal distribution with a mean value of *I*_μ_, with the degree of heterogeneity in the input currents determined by the standard deviation, σ. The values of *I*_μ_ range from 100 to 1,000 pA, and this value is varied in our heatmaps (described below) along the y-axis. σ = 3, 6 and 12 pA were studied.

### 2.3. Simulations

The code underlying these simulations was written in the C programming language and run on a Linux-based high-performance computing cluster utilizing Compute Canada resources provided via the University of Toronto (Loken et al., 2010). All simulations were run for 2,000 ms, with the initial conditions randomized such that *V*∈(−70, 0) while *u* = 0. Model equations were integrated using the Euler Method with a time step *dt* = 0.01 ms. Spikes did not trigger synaptic current until 100 ms into the simulation (via a simple manipulation in the code) to allow initial transients to decay.

In order to uncover other potential dynamical states of the network, a brief, large amplitude current pulse was delivered uniformly to each cell in the network to perturb the system and potentially bias it toward the synchronous dynamical state. This 2 ms pulse had an amplitude of 1,000 pA and was delivered at 1,000 ms, and is represented by the *I*_*perturb*_ term in Equation (1). This is analogous to imposing homogeneous initial conditions causing instantaneous spiking of all neurons in the network, in contrast to the randomized initial conditions that begin the simulations. To identify networks that exhibited bistability between asynchronous and clustered behavior, network dynamics established from random initial conditions (figure panels denoted *Random Initial Conditions*) and those established after the perturbation (figure panels denoted *Following Perturbation*) were compared.

Heatmaps of the Synchrony Measure (described below) and differences in the Synchrony Measure before and after the perturbation shown in all figures display the average of these scores over five independent simulations. The Random Initial Conditions scores were calculated based on the network activity from 500 to 1,000 ms, and the Following Perturbation scores were calculated based on the network activity from 1,500 to 2,000 ms. In the heatmap plots the mean applied current value *I*_μ_ was varied along the y-axis, while the inhibitory synaptic weight *g*_*syn*_ was varied along the x-axis. Simulations (not shown here) were run to ensure that the behaviors indicated by the Synchrony Measure taken over the given intervals were indicative of stable behaviors that would persist long past the time interval measured here.

### 2.4. Measures

The measure used to quantify coherent activity in the simulated networks, here termed a *Synchrony Measure*, is a slight adaptation of a commonly used measure created by Golomb and Rinzel (Golomb and Rinzel, 1993, 1994) that quantifies the degree of spiking coincidence in the network. This particular implementation of this measure has been utilized in previous studies (Rich et al., 2016, 2017, 2018).

Briefly, the measure involved convolving a Gaussian function with the time of each action potential for every cell to generate functions *V*_*i*_(*t*). The population averaged voltage *V*(*t*) was then defined as $V\left(t\right)=\frac{1}{N}\overset{N}{\underset{i=1}{\sum }}V_{i}\left(t\right)$, where *N* is the number of cells in the network. The overall variance of the population averaged voltage σ and the variance of an individual neuron's voltage σ_*i*_ were defined as

(6) $$\begin{array}{r} \sigma =<V{\left(t\right)}^{2}>-<V\left(t\right){>}^{2} \end{array}$$

and

(7) $$\begin{array}{r} \sigma_{i}=<V_{i}{\left(t\right)}^{2}>-<V_{i}\left(t\right){>}^{2} \end{array}$$

where < ·> indicates time averaging over the interval for which the measure is taken. The Synchrony Measure *S* was then defined as

(8) $$\begin{array}{r} S=\frac{\sigma }{\frac{1}{N}\sum_{i=1}^{N}\sigma_{i}} \end{array}$$

**Figure 3:**
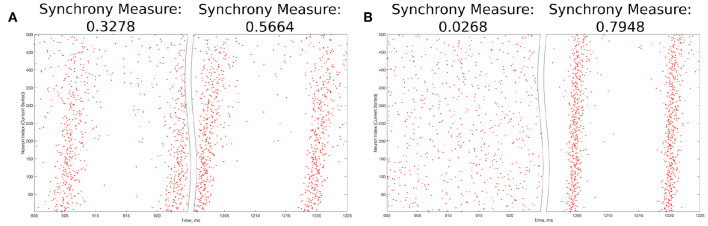
Example raster plots illustrating the Synchrony Measure and motivating the definition of the Bistability Measure. **(A)** An example network that exhibits “weak” synchronous dynamics both before and after the perturbation is delivered at 1,000 ms, resulting in a moderate value of the Synchrony Measure in each case. Dynamics before the perturbation are shown in the left panel, while dynamics following the perturbation are shown in the right. Although the Synchrony Measure following the perturbation is larger than that before the perturbation, this increase does not indicate a bistable transition from asynchronous to synchronous dynamics, but rather qualitatively “tighter” synchrony. The choice of 0.3 as the “threshold value” in the articulation of the Bistability Measure prevents cases such as this from contributing positively to the measure. **(B)** An example network exhibiting asynchrony before the perturbation (left panel) and very clear synchrony afterwards (right panel), along with the corresponding Synchrony Measures. Very low synchrony measures (typically <0.25) indicate asynchrony, while higher Synchrony Measures illustrate synchrony, with higher values indicating more structured, “tighter” synchrony.

The value *S* = 0 indicates completely asynchronous firing, while *S* = 1 corresponds to fully synchronous pattern of network activity. Example raster plots and the corresponding Synchrony Measure values over an illustrative range are shown in Figure 3.

This research was interested not merely in the degree of synchronous firing in the networks of interest (described by the Synchrony Measure), but rather was primarily focused on identifying a transition from asynchronous to synchronous dynamics driven by network bistability. A straightforward way to identify whether such a transition occurred following a perturbation (discussed in detail above) is to compare the value of the Synchrony Measure before and after said perturbation; in such a comparison, large increases in the Synchrony Measure following the perturbation are likely indicative of a transition from asynchronous to synchronous dynamics. Further, to analyze a network's predisposition toward this transition over a range of parameter values, one need only summate these individual comparisons in an informed fashion into a single, quantitative score. This motivated the creation of a *Bistability Measure* used in this study.

This measure was calculated in three steps. First, the difference between the Synchrony Measure following the modeled perturbation and the Synchrony Measure from randomized initial conditions was taken for each network in some context (i.e., for a particular parameter range encapsulated by a given heatmap). Second, only cases when this difference was >0.3 were included in the summation (this choice is justified in detail below). Finally, the sum of the Synchrony Measure differences exceeding this threshold value was taken to yield the final Bistability Measure.

The choice of the “threshold” value of 0.3 in the second step above merits further explanation. The Synchrony Measure is not a binary differentiation between asynchronous and synchronous dynamics, but rather a quantitative measure of the degree of synchronous firing. This means that increases in the Synchrony Measure, particularly subtle ones, do not necessarily indicate a differentiation of asynchrony from synchrony, but instead could indicate the presence of qualitatively “tighter” synchrony. An example of such a case is seen in Figure 3A. However, large increases in the Synchrony Measure are almost always indicative of entirely different dynamical states, as shown by the example in Figure 3B. After a thorough investigation of the correspondence between a qualitative assessment of synchrony (i.e., visual inspection of raster plots) and the quantitative assessment provided by the Synchrony Measure, it was determined that a difference of at least 0.3 in the Synchrony Measure before and after a perturbation best identified networks in which a transition between dynamical states occurred while excluding networks in which an increased Synchrony Measure only indicated subtle changes in the network dynamics.

Instantiating this “threshold” value into the calculation of the Bistability Measure ensures that the measure best quantifies the tendency for networks to exhibit bistable transitions, rather than naively quantifying the difference in Synchrony Measure before and after the perturbation. This occurs in two fashions during the calculation of the measure to further ensure robustness: first, networks that exhibit minor changes in the Synchrony Measure (<0.3) are completely excluded from the summation, considering such networks are extremely unlikely to exhibit a bistable transition; and second, the summation of the change in the Synchrony Measure values, rather than a binary summation of which networks exhibit a change above the threshold value, allows networks that exhibit a larger Synchrony Measure difference (for which one can much more confidently assert a dynamical transition occurs) to be weighted more heavily in the calculation of the Bistability Measure. Finally, the fact that this value was not chosen arbitrarily is worth further emphasis: this choice was made only after a detailed investigation into the interpretation of various Synchrony Measure differences and trial calculations of the Bistability Measure with various choices of this “threshold” value (not shown here), all of which that contained flaws improved upon by the choice made for the final measure.

### 2.5. Ornstein-Uhlenbeck Process

The perturbation described above is motivated primarily by the desire to uncover a mechanism for the transition from asynchrony to synchrony from the perspective of dynamical systems. In order to assess whether this mechanism is biologically reasonable, an analogous perturbation that might arise in more biologically grounded models was sought.

An Ornstein-Uhlenbeck process (Uhlenbeck and Ornstein, 1930) is used in the literature to model background synaptic input into a network (Destexhe et al., 2001; Piwkowska et al., 2008), and is used in this study to determine whether “perturbation-like” activity might arise naturally from this model of external synaptic input. This process, used to determine the conductance of excitatory synaptic input in this context, is described mathematically by the following equations (Destexhe et al., 2001) with an initial condition *g*_*e*_(0) = *g*_*e*0_:

(9) $$\begin{array}{r} g_{e}\left(t+h\right)=g_{e0}+\left[g_{e}\left(t\right)-g_{e0}\right]e^{-h/\tau_{e}}+A_{e}N\left(0,1\right) \end{array}$$

(10) $$\begin{array}{r} A_{e}=\sqrt{\left(\frac{D_{e}\tau_{e}}{2}\right)\left(1-e^{\frac{-2h}{\tau_{e}}}\right)} \end{array}$$

where *N*(0, 1) is a normal random number taken from a distribution with 0 mean and a standard deviation of 1.

The insights from Piwkowska et al. (2008) allowed for the choice of parameters constrained by cortical data. The parameters used in the Ornstein-Uhlenbeck process utilized in this study were *g*_*e*0_ = 3 nS, τ_*e*_ = 2 ms, and *D*_*e*_ = 2 (a unitless diffusion coefficient), and the integration time step was *h* = 0.01 ms.

The Ornstein-Uhlenbeck process motivates an alteration to the *I*_*perturb*_ current that will be described in more detail in the Results section.

### Code Accessibility

The code/software described in the paper is freely available online at https://github.com/FKSkinnerLab/CorticalInhibitoryNetwork.

## 3. Results

Synchronous interneuronal activity is implicated in oscillations representing both physiological and pathological brain states. Physiologically, the generation of ripples associated with sharp waves in the hippocampus is thought to be driven by a sudden onset of inhibitory synchrony caused by an increase in drive from CA3 (Schlingloff et al., 2014; Gulyás and Freund, 2015). Such data support the notion that a transition to oscillatory dynamics can be brought about by increased external drive, as was shown computationally (Ferguson et al., 2013; Rich et al., 2016). Hyperexcitability in inhibitory cells might represent an analog to this increased drive, potentially underlying the correlation between increased interneuronal activity and the synchronous GABAergic signaling observed prior to seizure or an inter-ictal spike (IIS). This hypothesis is consistent with the observation that interneuronal firing increases before pyramidal cell firing prior to IIS generation and seizures in animal epilepsy models (Lasztóczi et al., 2004, 2009; Gnatkovsky et al., 2008; Muldoon et al., 2015; De Curtis and Avoli, 2016; Miri et al., 2018), and in humans *in vivo* (Elahian et al., 2018).

In this work we sought an explanation for this increased interneuronal firing prior to seizure onset. We model the excitable state induced by 4-AP, a commonly implemented model system (Perreault and Avoli, 1991; Kibler and Durand, 2011; Williams and Hablitz, 2015; Baird-Daniel et al., 2017; Wenzel et al., 2017; Chang M. et al., 2018; Liou et al., 2018; Chang et al., 2019; Shivacharan et al., 2019) to study seizure dynamics, and in which we (Chang M. et al., 2018) have shown that interneurons are complicit in seizure onset.

### 3.1. Bistability Between Coherent and Incoherent States Is Exhibited More Robustly by 4-AP Inhibitory Networks

The concept of bistability arises primarily from the mathematical study of dynamical systems. In this context, a “stable” state is one which will be preserved by the system for all time in the absence of any perturbations to the conditions defining the system. In non-linear systems it is possible for multiple stable states to exist, and for the network to naturally settle into any one of these stable states depending upon a variety of factors including the initial conditions and any perturbations that might be delivered. In a biological system, this could manifest from the history of inputs from different brain structures along various pathways to the network in question. Such a system is defined to be “bistable” or “multistable” given the existence of more than one stable solution to the mathematical equations (Izhikevich, 2007).

**Figure 4:**
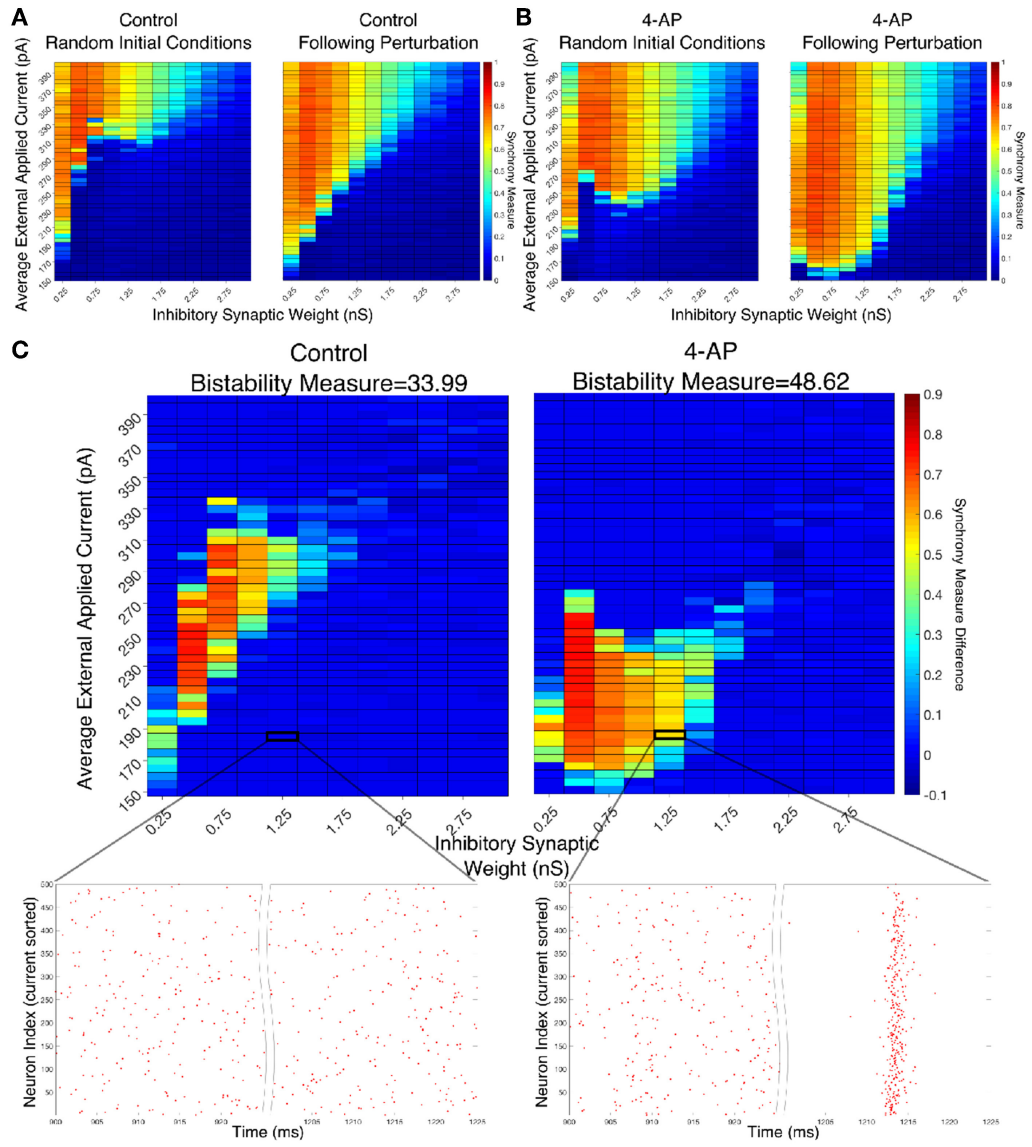
Networks containing model 4-AP neurons are more prone to bistability than networks of control neurons. **(A,B)** Heatmaps displaying the Synchrony Measure for control networks **(A)** and 4-AP networks **(B)** with a connectivity probability of 0.12 and a standard deviation amongst the driving currents of 6 pA. In these heatmaps, the inhibitory synaptic weight is varied along the x-axis and the average external applied current is varied along the y-axis. The left panel displays the measure taken from randomized initial conditions, while the right panel displays the measure taken after a modeled perturbation. **(C)** Heatmaps over the same parameter regime, but now showing the difference between the Synchrony Measure shown in the right and left heatmaps in **(A,B)**. Control results are shown on the left, and 4-AP results are shown on the right. 4-AP model networks are much more likely than control model networks to exhibit a change in dynamics following the perturbation (as indicated both by more warm colors in the heatmap and by the increased Bistability Measure score shown above the panels), indicating that the perturbation induced a transition from asynchronous to synchronous dynamics indicative of a bistability. A raster plot for both the control and 4-AP settings for a network with an inhibitory synaptic weight of 1.25 nS and an average external applied current of 185 pA (corresponding to the outlined box in the heatmap) is shown, providing an illustrative example of a case where the transition from asynchronous to synchronous dynamics following the perturbation, and thus the existence of a bistability, is observed in the 4-AP but not the control case.

The results presented in Figure 4 show that many of the networks within the parameter regime considered in this work exhibit bistability. In Figures 4A,B the Synchrony Measure (described in the Materials and Methods section) was taken for the same networks in two different states: the results from randomized initial conditions are shown in the left panels, while the results following a perturbation to the system (described in the Materials and Methods section) are shown in the right panels. Note that the parameter range shown in these heatmaps is “zoomed in” relative to the larger parameter scan used in the heatmaps presented in the following section in order to better highlight the regime of bistability. Control networks are shown in Figure 4A while 4-AP networks are shown in Figure 4B.

There appear to be a number of networks in both the control and 4-AP settings that show a high Synchrony Measure, and thus coherent network states, following the perturbation but not from randomized initial conditions. This is indicative of a bistable system in which both the coherent and incoherent states are stable, even though the network might require a perturbation in order to leave the incoherent stable state and settle into the coherent stable state. This result is highlighted by Figure 4C in which the difference between the Synchrony Measure following the perturbation and the Synchrony Measure from randomized initial conditions is plotted to highlight the networks in which this difference occurs. Qualitatively, it appears not only that the parameter regime including these type of networks is shifted when comparing the 4-AP and control cases, but most importantly it appears that more of these types of networks exist in the 4-AP setting as opposed to the control case. To quantify this observation, a Bistability Measure (as outlined in the Materials and Methods section) was used, revealing that, indeed, the parameter regime defining bistable networks is larger in the 4-AP case. Raster plots highlighting an example network that is bistable in a 4-AP network, but not in the control case, for the same parameter values are shown below these heatmaps.

It makes sense, in the context of the study of seizure, that both control and 4-AP networks would exhibit some bistability. Indeed, it is well-established that all brains are capable of generating a seizure, even though seizures are much more likely in individuals with epilepsy (see the literature on seizures arising in non-epileptic patients following traumatic brain injury Verduyn et al., 1992; Schierhout and Roberts, 2001). However, it is interesting in the context of the increased interneuronal activity observed prior to seizure onset that 4-AP networks were more likely to exhibit bistability than control networks. This result supports the hypothesis that 4-AP treated, hyperexcitable interneurons are more likely to be vulnerable to a mechanism increasing the overall firing rate of inhibitory neurons, which in this context is the transition from asynchronous to synchronous firing via a “bistable transition” (the connection between synchrony and increased firing rate is described in detail below). It is also interesting to note that the bistable regime is both wider (i.e., encompassing a larger range of synaptic strengths) and includes lower driving currents for 4-AP networks, although the latter is perhaps expected due to the lower rheobase of 4-AP neurons.

**Figure 5:**
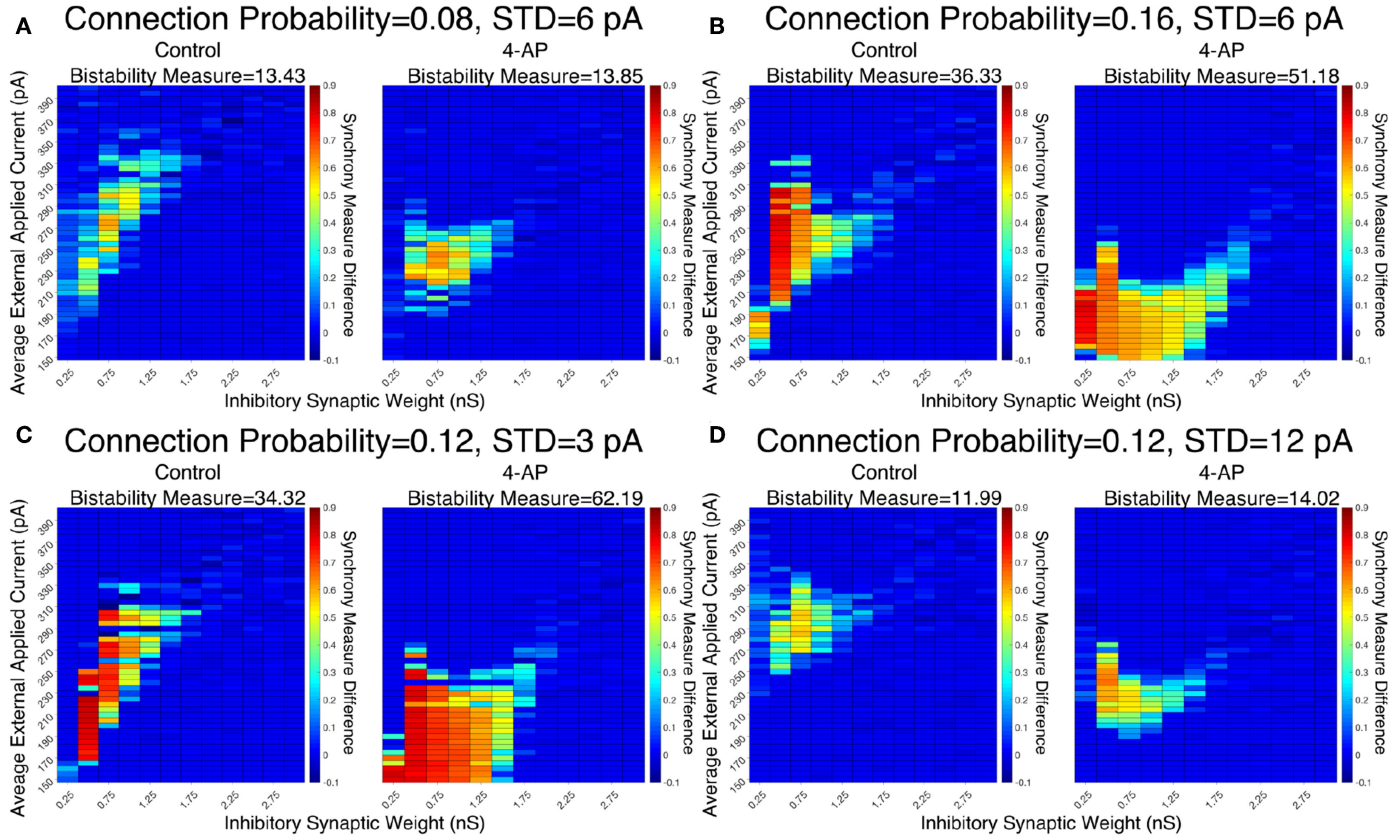
Networks containing model 4-AP neurons exhibit bistability more robustly than control networks for a variety of network parameters. **(A–D)** Heatmaps displaying the difference between the Synchrony Measure following a modeled perturbation and from randomized initial conditions for control networks (left) and 4-AP networks (right). In these heatmaps, the inhibitory synaptic weight is varied along the x-axis and the average external applied current is varied along the y-axis. The Bistability Measure for each condition is shown above the corresponding panel. Results for a connection probability of 0.08 and standard deviation of 6 pA are shown in **(A)**, results for a connection probability of 0.16 and standard deviation of 6 pA are shown in **(B)**, results for a connection probability of 0.12 and standard deviation of 3 pA are shown in **(C)**, and results for a connection probability of 0.12 and standard deviation of 12 pA are shown in **(D)**. In all cases, 4-AP model networks exhibit a larger parameter regime showing behaviors indicative of a bistability than analogous control networks, shown both by more warm colors in the heatmap and the increased Bistability Measure.

The robustness of this result was confirmed when networks were subjected to different degrees of heterogeneity in the external driving currents and different connection probabilities. This is shown by the Synchrony Measure difference heatmaps and Bistability Measures shown in Figure 5. Indeed, in all four cases presented (varying connection probability in Figures 5A,B and varying standard deviation of the external applied currents in Figures 5C,D, the 4-AP networks were more likely to exhibit bistability, as seen via a joint analysis of the Bistability Measures and the bistable parameter regime in the heatmaps.

The analysis of these *in silico* networks through the lens of the mathematical concept of bistability reveals crucial properties of 4-AP networks that could not otherwise be identified. However, the question remains whether a transition of this type is biologically feasible, especially considering the perturbation used to reveal the existence of the bistability was motivated from dynamical systems insights rather than the underlying biology. We address this using an Ornstein-Uhlenbeck process [as described in the Materials and Methods section (Destexhe et al., 2001; Piwkowska et al., 2008)] to generate a reasonable approximation of background excitatory synaptic conductance in the cortex. Such synaptic activity can be thought of as a more biologically-grounded analog for the *I*_*app*_ tonic driving current used in the computational models here. The conductance generated by the Ornstein-Uhlenbeck process is transformed into a driving current simply by multiplying by (*V*−*E*_*syn*_), where here *E*_*syn*_ takes on an excitatory value of 0 mV and *V* is approximated as the resting potential of the neuron (here −60.6 mV).

**Figure 6:**
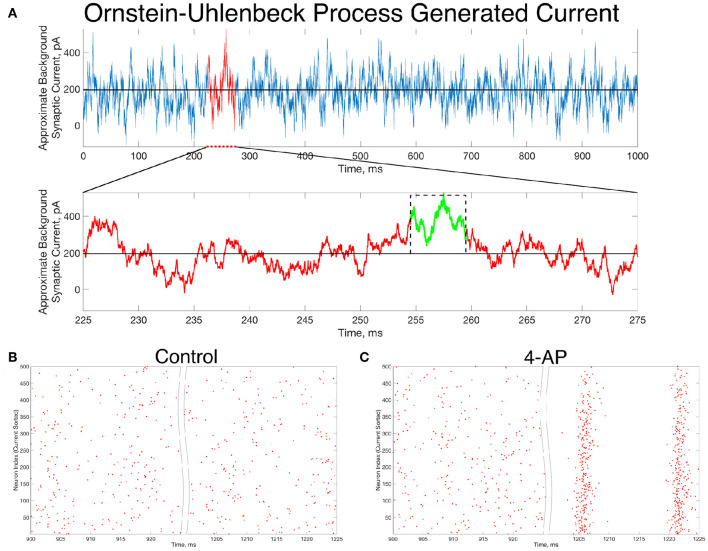
*In vivo*-like excitatory background synaptic currents can also elicit “bistable transitions” in the model inhibitory networks. **(A)** An example of excitatory background synaptic current (1,000 ms in the top panel) generated using an Ornstein-Uhlenbeck Process with parameters informed by cortical experimental literature. The bottom panel zooms in on a region of interest (plotted in red) revealing a brief period (plotted in green) in which the current is significantly larger than its average value, activity which has perturbation-like qualities. This activity is approximated by a current pulse of similar width and amplitude, plotted on the figure in a dashed black line. **(B,C)** Raster plots for a control **(B)** and 4-AP **(C)** network that is identical to the examples displayed in Figure 4, where the large, brief current pulse used as the perturbation throughout this study is replaced by a current pulse informed by the Ornstein-Uhlenbeck Process shown in **(A)** that represents *in vivo*-like activity. Despite this change, which amounts to a wider pulse with significantly lower amplitude, the control and 4-AP networks still exhibit antithetical responses to this perturbation; namely, control networks return to asynchronous firing following the perturbation while 4-AP networks transition into synchronous dynamics.

An example of such a current, generated for 1,000 ms, is seen in Figure 6A. Zooming in on the red portion of the current (225–275 ms), a 5 ms portion of the current trace that retains a significantly higher than average value is highlighted in green. This current is simplified for computational implementation by a square current pulse with an amplitude of 320 pA and a 5 ms duration, approximated on the figure with a dotted black line. By utilizing this square pulse as our perturbation delivered 1,000 ms into the simulation, represented in Equation (1) by the *I*_*perturb*_ term, we can investigate whether a less idealized perturbation that is more reasonable based on *in vivo* activity might still drive the transition from asynchrony to synchrony.

Indeed, the “bistable transition” typified by the raster plots in Figure 4 is preserved when the perturbation is replaced by the current pulse motivated by the results from the Ornstein-Uhlenbeck process, as shown in Figures 6B,C. This result suggests that a “bistable transition” is viable in a more biologically-grounded setting, as it can be triggered by a perturbation that could reasonably occur due to fluctuations in the background excitatory synaptic activity in the cortex. Taken together with the detailed analysis presented above of the bistability present in these networks from the perspective of dynamical systems, it is apparent that a transition from asynchrony to synchrony in inhibitory networks caused by a “bistable transition” is both a computationally and biologically plausible mechanism explaining the corresponding activity observed experimentally prior to seizure.

### 3.2. Transitions From Asynchrony to Synchrony in Inhibitory Networks Correspond With an Increase in Firing Frequency

**Figure 7:**
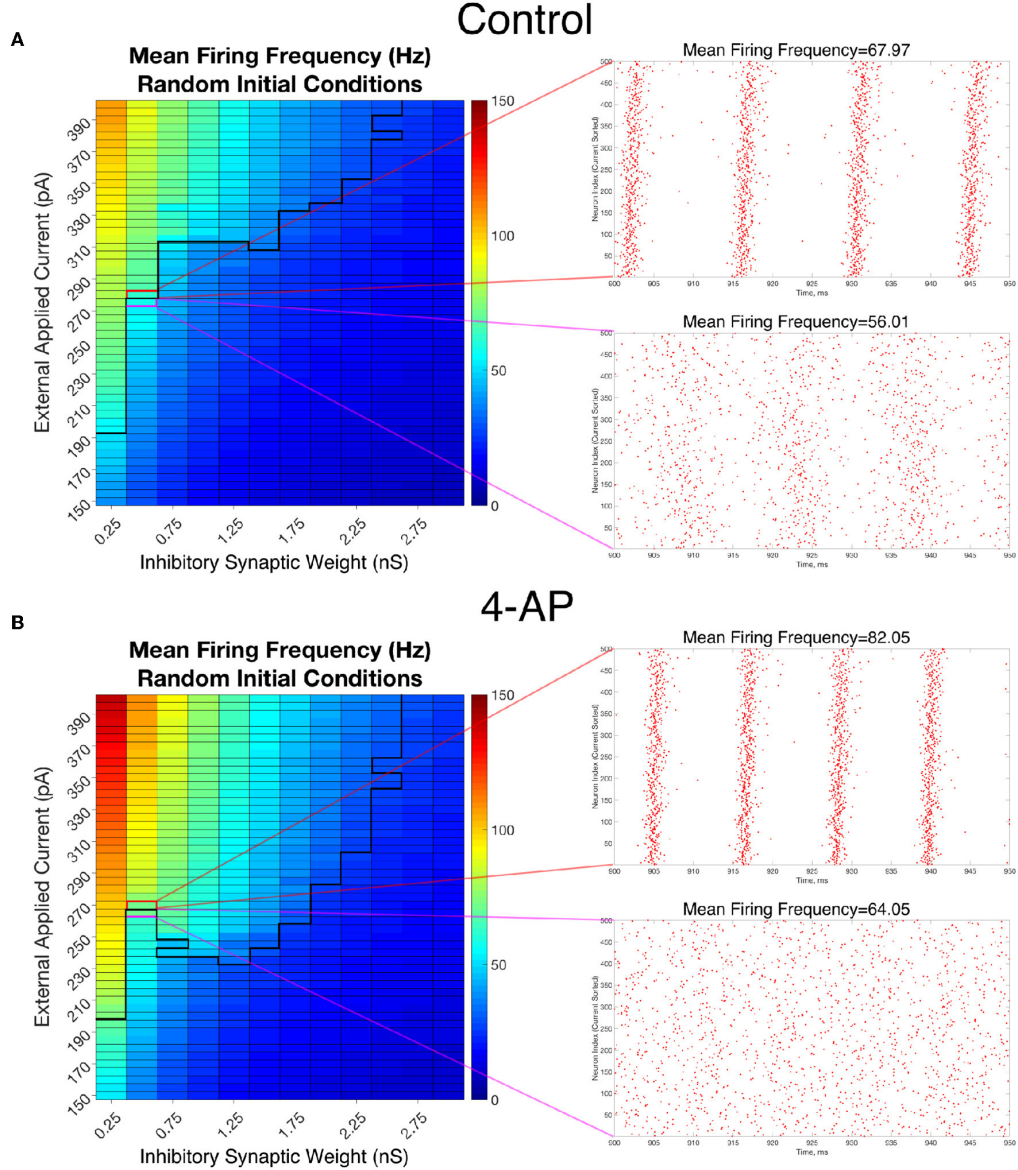
Overall firing frequency is higher in networks exhibiting synchrony, relating the transition from asynchrony to synchrony to increased interneuronal activity. **(A,B)** Mean Firing Frequency values, averaged over five independent simulations, for control **(A)** and 4-AP **(B)** networks. The border dividing the parameter regime supporting synchronous dynamics (top-left) from the regime of asynchrony is depicted by the bolded black line, where this border was found using a cutoff value for the Synchrony Measure of 0.25 (which was found to be reasonable after a rigorous investigation of a variety of raster plots and their corresponding Synchrony Measures). Example raster plots for the networks outlined in pink and red illustrate example asynchronous and synchronous raster plots, respectively, along this border. Their Mean Firing Frequency values illustrate the relatively large increase in network activity that is associated with the transition from asynchronous to synchronous firing.

To analyze the firing rate of our networks, a “Mean Firing Frequency” measure (which involves simply summing the total number of spikes in the network over a given time interval, dividing by the number of cells, and then converting this value into a frequency by dividing by the length of the time interval) was taken over the last 500 ms of simulations performed from random initial conditions over the parameter space used in Figure 4. Critically, this analysis reveals that both control and 4-AP networks show a similar increase in average firing rate when transitioning from asynchrony to synchrony (highlighted by the example raster plots and corresponding mean firing frequencies presented in Figure 7). This result is analogous to a similar finding in previous work on inhibitory networks (Ferguson et al., 2013) and indicates that the transition into synchrony described by the “bistable transition” mechanism corresponds with an increase in overall inhibitory cell activity, as seen *in vivo* and *in vitro* (Lillis et al., 2012; Muldoon et al., 2015; Elahian et al., 2018). Thus, bistability explains why 4-AP networks are more prone to transition from asynchrony to synchrony than their control counterparts, and also why 4-AP treated networks are more vulnerable to the increased interneuronal activity observed prior to seizure onset.

The results in Figure 7 also reveal that the average cell firing frequency in control and 4-AP networks with similar network parameters and similar dynamical states (i.e., synchrony or asynchrony) are actually quite close (and any differences are certainly diminished from the extreme differences seen in their FI curves presented in Figure 2). This finding is fairly robust over all but the weakest inhibitory synaptic weights. This implies that the mechanism involved in the “bistable transition” involves an interplay of cellular (potentially not only the hyperexcitability, but also the increased adaptation, in 4-AP neurons) and network properties, and could not be replicated merely by causing the neurons to fire faster in some artificial fashion. Indeed, it does not appear that the firing rate itself serves a disproportionate role in dictating the overall network dynamics.

We note that this finding provides a potential avenue for an experimental exploration of the results presented here: namely, a substantial increase in firing rate following a perturbation to an inhibitory network is likely indicative of a transition into synchronous firing. Such behavior is likely more easily identifiable by multi-electrode arrays than synchronous firing itself.

Furthermore, these results shed new light on the interaction between synchrony and increased cell firing rate in the context of pre-ictal neuronal activity. Experimental literature commonly shows that these dynamics (in both excitatory and inhibitory cells) both accompany seizure onset (see, for example, the work in humans of Schevon et al., 2012), with many of these studies implying that increased firing rate plays a causal role in the transition into synchrony (see, for example, the work of Elahian et al., 2018 which reveals an increase in interneuron firing rates prior to seizure and the corresponding synchronous dynamics). However, the “bistable transition” described in this paper does not require a change to the system that would increase the average cell firing rate; rather, the increased firing rate comes about seemingly driven by the induced synchronous firing of the inhibitory network. Thus, it is possible that synchrony of inhibitory networks is permissive of an increased neural firing rate, instead of increased firing rate causing this synchrony. Indeed, where there is sparse sampling of interneurons, increased firing rates of interneurons prior to a seizure may be additionally interpreted from our modeling results to represent a transition to synchronous interneuronal firing (Elahian et al., 2018) rather than a firing rate increase alone.

### 3.3. Sharp Transitions Between Coherent and Incoherent States Caused by Increased External Input Are Unlikely to Underlie Interneuronal Hyperactivity Prior to Seizure Onset

In CA1 hippocampal inhibitory network models constrained in size, connection probability, cellular and synaptic properties, Ferguson et al. (2013) demonstrated “sharp transitions” between asynchronous and synchronous firing caused by a small, permanent increase in the external drive to the network. This “increased drive” mechanism has both experimental and computational support (see the discussion in the beginning of the Results section) for explaining a transition into synchrony in purely inhibitory networks. Given the correspondence between inhibitory network synchrony and increased firing rate discussed above and observed in this previous work (Ferguson et al., 2013), we investigated “increased drive” as a potential mechanism explaining the increased firing rate of interneurons observed experimentally prior to seizure. Indeed, potentially eliminating “increased drive” as a candidate mechanism would provide additional support for the viability of the “bistable transition” mechanism described above.

**Figure 8:**
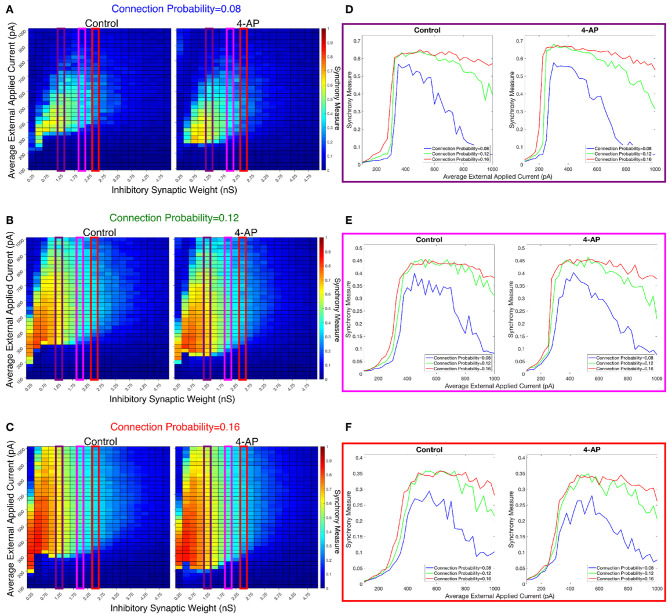
Cortically motivated inhibitory networks exhibit a “sharp transition” between asynchronous and synchronous dynamics driven by an increase in the external drive for various connection probabilities. **(A–C)** Heatmaps displaying the Synchrony Measure for control networks (left) and 4-AP networks (right) with a standard deviation amongst the driving currents of 6 pA and varying connectivity densities. In these heatmaps, the inhibitory synaptic weight is varied along the x-axis and the average external applied current is varied along the y-axis, and the measure is taken from random initial conditions. **(D–F)** Two dimensional “slices” of the heatmaps in **(A–C)** taken to better illustrate the sharpness of the transition between asynchronous and synchronous dynamics as well as more easily compare this sharpness both across varying connection probabilities and between control and 4-AP conditions. **(D)** Shows results for an inhibitory synaptic weight of 1.25 nS, **(E)** for an inhibitory synaptic weight of 2.0 nS, and **(F)** for an inhibitory synaptic weight of 2.5 nS. There is no major difference in the tendency for 4-AP vs. control networks to exhibit the “sharp transition” from asynchrony to synchrony despite differences in the parameter regime supporting synchrony. Furthermore, the differences in the “sharpness” of the transition in the two cases are not robust.

We investigated the tendency for inhibitory networks of both control and 4-AP neurons to synchronize from randomized initial conditions with varying connection probabilities and levels of heterogeneity. Figure 8 shows results illustrating network coherence for a parameter scan over a range of inhibitory synaptic strengths that encompass physiological estimates (Markram et al., 2015) and average external applied currents with varied connection probabilities.

**Figure 9:**
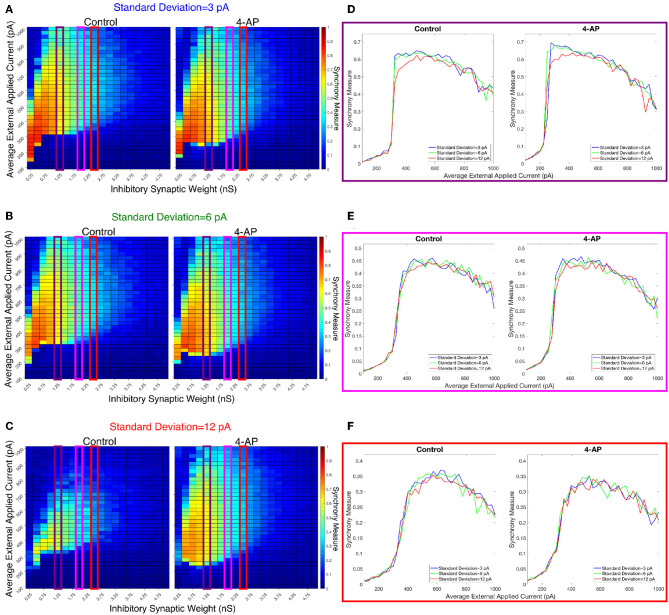
Varying the heterogeneity in external driving current in modeled purely inhibitory networks largely preserves the general dynamical differences and similarities seen between the 4-AP and control cases from randomized initial conditions. **(A–C)** Heatmaps displaying the Synchrony Measure for control networks (left) and 4-AP networks (right) with a connection probability of 0.12 and varying standard deviations amongst the driving currents. In these heatmaps, the inhibitory synaptic weight is varied along the x-axis and the average external applied current is varied along the y-axis, and the measure is taken from random initial conditions. **(D–F)** Two dimensional “slices” of the heatmaps in **(A–C)** taken to better illustrate the sharpness of the transition between asynchronous and synchronous dynamics as well as more easily compare this sharpness both across varying connection probabilities and between control and 4-AP conditions. **(D)** Shows results for an inhibitory synaptic weight of 1.25 nS, **(E)** for an inhibitory synaptic weight of 2.0 nS, and **(F)** for an inhibitory synaptic weight of 2.5 nS. Once again, there is no significant difference in the tendency for 4-AP vs. control networks to exhibit the transition from asynchrony to synchrony, nor any significant differences in the “sharpness” of this transition.

Figures 8D–F show three two-dimensional plots highlighting the evolution of the Synchrony Measure as a function of the average external applied current for a set value of the inhibitory synaptic weight. Results for each connection probability are shown jointly to facilitate comparison, with results for control networks shown in the left panels and results for 4-AP networks shown in the right panels. Additionally, the “sharpness” of the transition from asynchrony to synchrony was quantified by taking the slope of the line segment best representing this transition, which is chosen to be that between the first point that achieves a Synchrony Measure greater than half the maximum Synchrony Measure observed by networks in that panel and the point one current step earlier. The slopes for all of the examples presented in Figures 8, 9 are shown jointly in Table 2.

**Table 2:** Slopes quantifying “sharpness” of the transition from asynchrony to synchrony seen in Figures 8, 9.

**Connection probability**	**Standard deviation (pA)**	**Inhibitory synaptic weight (nS)**	**Control slope**	**4-AP slope**
		1.25	0.0125	**0.0135**
0.08	6	2.00	**0.0059**	0.0047
		2.50	**0.0030**	0.0012
		1.25	**0.0204**	0.0172
0.12	3	2.00	0.0074	**0.0096**
		2.50	**0.0041**	0.0033
		1.25	0.0191	**0.0211**
0.12	6	2.00	0.0038	**0.0057**
		2.50	0.0027	**0.0042**
		1.25	0.0135	**0.0138**
0.12	12	2.00	0.0043	**0.0074**
		2.50	0.0034	**0.0039**
		1.25	0.0106	**0.0162**
0.16	6	2.00	0.0059	**0.0108**
		2.50	0.0027	**0.0037**

*For each parameter set, the larger slope is bolded for ease of reference*.

The results presented in Figure 8 show that a sharp transition between asynchrony and synchrony caused by a small, permanent increase in external driving current does occur in these cortically-motivated networks over a range of reasonable connection probabilities, both for control and 4-AP neurons. As one would intuitively expect, the parameter regime in which network coherence occurs grows larger as the connection probability becomes larger (Figures 8A–C). The two-dimensional plots (Figures 8D–F) do not show a clear pattern between the connection probability and the sharpness of the transition, but this is reasonable considering that large connection probabilities were not included in our explorations (see Methods). The differences between control and 4-AP networks were also observed when the heterogeneity was varied as shown in Figure 9. Heatmaps analyzing the Synchrony Measure over the entire parameter regime are shown in Figures 9A–C, with similar comparison between control and 4-AP networks as in Figure 8, while analogous two-dimensional plots to those in Figure 8 are shown in Figures 9D–F, but with varying standard deviations as opposed to connection probabilities in each panel. The results presented in Figure 9 show the expected effects of increased heterogeneity: namely, as the heterogeneity increases, the size of the parameter regime exhibiting coherent states decreases. This is shown most clearly by comparing the results with a standard deviation of 12 pA to both the results with a standard deviation of 3 and 6 pA, which show similar regimes of synchronous dynamics (although the synchrony is more pronounced over this regime when the heterogeneity is smallest at 3 pA).

While these results show the existence of transitions caused by increased external drive that are “sharp,” Figures 8, 9 do not reveal any difference in the tendency for control or 4-AP networks to exhibit this sharp transition. While 4-AP networks exhibit a high synchrony measure over a wider parameter regime, particularly at lower values of the average external applied current (explained by a combination of the hyperexcitability of 4-AP neurons, insights from the ING mechanism, and the analysis of sparsely connected inhibitory networks presented by Rich et al., 2016), this does not indicate an increased tendency to exhibit the *transition* from asynchrony to synchrony. Indeed, such a transition exists almost uniformly across the inhibitory synaptic weights studied here (which can be seen both by visually inspecting the increase in Synchrony Measure going up a column in the heatmaps or looking at the two-dimensional traces), with only the applied current value at which the transition occurs changing.

Furthermore, any comparisons of the relative “sharpness” of the transitions in control and 4-AP networks are qualitative at best. In a majority of the comparisons illustrated in the two-dimensional plots (Figures 8D–F), 4-AP networks displayed a higher slope measure shown in Table 2 than their control counterparts. However, this feature is not entirely robust (see, for example, the comparison of networks with a connection probability of 0.08 in Figure 8E and Table 2), and there is no guarantee that the minor increases in the slope measure are indicative of a difference in the actual dynamics underlying the transition. Further detailed analysis of this feature of the networks would be required to draw any conclusions.

Finally, it is worth noting that a change of this sort is unlikely to arise via an Ornstein-Uhlenbeck process modeling background excitatory synaptic activity, in contrast to what was shown in Figure 6 (i.e., that perturbation-like activity could arise from this process). Indeed, the example current presented in Figure 6 does not show any large amplitude increases in the synaptic current lasting longer than tens of milliseconds. This indicates that a small, permanent increase to the external drive to the network likely requires a more consequential biological change in the system, especially when compared to a perturbation which can arise more naturally via fluctuations in the background synaptic activity.

Taken together, these results confirm that a transition from asynchrony to synchrony as a result of minor, permanent increases to the external driving current can occur in these cortically-motivated networks, similar to the results presented by Ferguson et al. (2013) in the hippocampus. However, there is no robust difference in the tendency for 4-AP vs. control networks to exhibit this transition. This strongly suggests that this mechanism is unlikely responsible for the increase in interneuronal activity seen prior to seizure. Instead, a mechanism driven by a “bistable transition” more plausibly explains seizure-related interneuronal dynamics, as this mechanism is much more likely to occur in 4-AP networks rather than control networks. While transitions into synchrony caused by minor, permanent increases to the external drive to an inhibitory network certainly could occur in the brain given the existing literature, this conclusion implies that the “increased drive” mechanism is more likely to underlie non-pathological oscillations rather than the pathological inhibitory synchrony potentially initiating seizure.

## 4. Discussion

Computational models at various scales and levels of detail have proven pivotal to our understanding of various aspects of seizure (Wendling et al., 2016). Here, we use such techniques to provide a putative *in silico* mechanism explaining how increased interneuronal activity, as well as interneuronal synchrony, might come about in a pro-ictogenic setting driven by network bistability. Our results provide preliminary mechanistic support for the viability of early steps in the multi-stage “GABAergic initiation hypothesis” of seizure. This hypothesis proposes that synchronous activation of inhibitory interneurons is the inciting event in an “all-or-none” phenomenon (Chang M. et al., 2018) which leads to a cascade of events resulting in a seizure. The immediate consequence of a sudden bolus of inhibitory drive is the generation of rebound spiking in pyramidal cells, that then feeds back on the inhibitory neurons resulting in a positive feedback loop and seizure initiation (Figure 1).

In this study, inhibitory networks informed by experiments in cortical interneurons in control and hyperexcitable settings were constructed, with the modeled hyperexcitability specifically mimicking the treatment of interneurons with 4-AP. Experimentally, *in vivo* and *in vitro* treatment with 4-AP induces seizures that are preceded by interneuronal synchrony and predominantly GABAergic IIS (Grasse et al., 2013; Muldoon et al., 2015). Thus, 4-AP is a commonly used model to study acute ictogenesis (Perreault and Avoli, 1991; Kibler and Durand, 2011; Williams and Hablitz, 2015; Baird-Daniel et al., 2017; Wenzel et al., 2017; Chang M. et al., 2018; Liou et al., 2018). Although 4-AP induces a multitude of effects, we focused on modeling the induced hyperexcitability given that this feature is thought to be the primary mechanism underlying the pro-ictogenic nature of this compound (Chang et al., 2019; Shivacharan et al., 2019).

GABAergic activity appears to not only play a role in seizure initiation under 4-AP conditions, but in other seizure models as well. Synchronous interneuronal activation has also been shown to underlie IIS in the *in vivo* pilocarpine model of epilepsy (Muldoon et al., 2015), precede seizures in both the low-Mg, high K^+^ model (Lasztóczi et al., 2004, 2009) and electrical stimulation models of seizure initiation (Velazquez and Carlen, 1999), and more generally precede seizures in rodents (Avoli and de Curtis, 2011; Muldoon et al., 2015). Thus, given the ubiquity of the increased inhibitory neuronal activity directly preceding seizure onset, insights gained from this study are likely translatable to the general study of neural dynamics prior to the initiation of epileptiform activity.

We examined two potential mechanisms by which inhibitory networks could suddenly transition into synchronous firing, and in turn increase the overall interneuronal firing rate. The mere existence of such a transition was not of primary interest in this study; rather, we focused on identifying whether these transitions occurred appreciably more often in hyperexcitable (i.e., 4-AP treated) networks when compared to control networks. Indeed, for a mechanism to be a viable candidate explaining dynamics occurring primarily in pre-ictogenic systems, it should intuitively be more likely to occur in a pathological, as opposed to healthy, setting. One potential mechanism previously presented Ferguson et al. (2013) and confirmed by other studies for more general networks (Rich et al., 2016), proposes that small, permanent increases in excitatory drive could cause a sharp transition between incoherent and coherent states in a purely inhibitory network. While transitions of this type were present in the networks studied here, there was no difference in the tendency for this transition to occur when 4-AP and control networks were compared. In contrast, transitions caused by a brief perturbation to external drive to the system, termed “bistable transitions,” were notably more likely to occur in 4-AP than control networks. This crucial difference implies that “bistable transitions” are a more viable candidate mechanism that explains the dynamics of inhibitory neurons seen in pathological networks.

The general concept of bistability has been discussed previously in epilepsy literature, given that epilepsy as a disease represents the sudden transition between two seemingly stable brain states: the “healthy” non-seizure state characterized by largely uncorrelated neural activity and the “pathological” seizure state characterized by synchronous neural firing (Da Silva et al., 2003). However, in this work, the setting in which bistability is analyzed is unique: we seek to explain a shift in inhibitory network dynamics that is observed experimentally and potentially might affect the behavior of the network in the moments prior to seizure onset. We do not assert that the bistability studied here, nor the mechanism described in this work, represents seizure initiation itself. Indeed, existing studies investigate a bistability between seizure and non-seizure states in settings, such as intact hippocampal slices (Chang W. et al., 2018), a computational network of both excitatory and inhibitory cells with special emphasis on the role of extracellular potassium concentrations (Fröhlich et al., 2010), or more general mathematical settings (Da Silva et al., 2003). In contrast, in this study bistability is analyzed solely in an inhibitory network, and the bistability does not in itself represent the transition into seizure, but rather a dynamical change that might precipitate seizure onset due to its downstream effects (as illustrated by a “GABAergic initiation hypothesis” schematized in Figure 1).

We also highlight an important distinction between this work and other computational work investigating the role of GABAergic signaling in epileptiform activity and inter-ictal discharges (IID): while recent literature investigating this topic makes use of the potential depolarizing capacity of GABA (Chizhov et al., 2017, 2019), the work presented here uses purely inhibitory GABAergic synapses. Indeed, while changes in the GABA reversal potential are seen during seizure *propagation* (Ellender et al., 2014), the changes in chloride concentrations necessary to elicit this feature do not exist prior to or during seizure *initiation* (Ellender et al., 2010; Chang M. et al., 2018), which is the focus of this research. Moreover, the mechanisms proposed in the work of Chizhov et al. (2017, 2019) that investigate the potential causal role of GABA in seizure initiation do not focus on the capacity of excitatory cells for PIR, in contrast to the “GABAergic initiation hypothesis” discussed here.

### 4.1. Details of the “Bistable Transition” and “Increased Drive” Mechanisms

The exploration of a transition driven by a small, permanent increase to the external drive was motivated by modeling studies (Ferguson et al., 2013) and physiological evidence (Schlingloff et al., 2014) of inhibitory networks in the hippocampus. The observed sharp transition in the hippocampal model networks of Ferguson et al. (2013) was dependent on constraining the model network from cellular, synaptic and connectivity perspectives with the experimental data and context. The research presented here reveals that those hippocampal insights were translatable to a more generic, cortically-motivated network. It is thus possible that our findings are generalizable to most fast-firing inhibitory networks, although parameters representing external drive and synaptic strengths would not necessarily be the same. Additionally, considering the similarities in neural and network properties utilized in this work and that of Ferguson et al. (2013), it is very probable that the hippocampal networks would exhibit bistability of some form. However, of critical importance in the context of this study is the lack of an appreciable difference in the tendency for 4-AP and control model networks to exhibit this transition.

The “bistable transitions” mechanism articulated in this paper addresses the shortcomings, in the context of seizure initiation, of the “increased drive” mechanism (Ferguson et al., 2013). Bistability arises on a small scale in many neuron models, including the Hodgkin-Huxley equations, in which both the resting state and periodic firing of action potentials are stable solutions and the amplitude of the input to the system determines which of these dynamics is exhibited by the model (Izhikevich, 2007). Here, we observed bistability on a larger scale, between network dynamics of coherent and incoherent network states. These states were uncovered by making use of a perturbation utilized previously in a more abstract study of inhibitory networks (Rich et al., 2016). Critically, the transition from asynchrony to synchrony brought about by this idealized perturbation (motivated from a mathematical perspective to reveal any potential bistability) persisted when a significantly less idealized perturbation (motivated by activity that might arise from an Ornstein-Uhlenbeck process simulating background excitatory synaptic activity) was used. This result indicates that this transition is potentially viable in a biologically-grounded setting as well.

The analysis of this “bistable transition” reveals that it is more likely to occur in 4-AP networks as opposed to their control counterparts. While the exact mechanism for the expansion of this bistable regime was not the focus of this work, we note that previous investigations of this bistability by Rich et al. (2016) revealed that this transition was driven by an interaction between the “phase-resetting” properties of the modeled interneurons' Type I phase response curves and the impetus of ING-driven inhibitory synchrony. Interestingly, the PRCs of our 4-AP interneurons exhibit stronger “phase-resetting” characteristics in comparison to the control interneuron, serving as a potential mechanistic explanation of the differential predisposition toward bistable transitions in these networks. However, the more detailed mathematical analysis required to support this hypothesized mechanism is outside the focus of this work, although it provides an interesting avenue for further research.

Nonetheless, this result indicates that bistability is a much more likely culprit in the initial step of a “GABAergic initiation hypothesis” of seizure than a transition brought about by a small, permanent increase in external drive to the network. Moreover, the physiologically-motivated Ornstein-Uhlenbeck process input current displayed in Figure 6 illustrates that perturbation-like activity is more likely to arise from background synaptic excitation than longer-lasting increases approximating a permanent increase in the external drive to an inhibitory network. Taken together, these insights support that dynamical changes made possible by network bistability to explain how interneuronal populations are “hijacked” in pathology (Beenhakker and Huguenard, 2009).

Finally, we note that in both mechanisms investigated here, the transition from asynchrony to synchrony is associated with increased firing rates. This not only justifies our investigation into network synchrony in the context of explaining increased interneuronal firing prior to seizure, but also helps to reconcile experimental evidence showing both increased interneuronal firing and interneuronal synchrony prior to seizure onset. This relationship is a particularly fertile ground for future experimental research.

### 4.2. The Relationship Between the “Bistable Transition” Mechanism and More Theoretical Computational Studies of Inhibitory Oscillations

The multi-scale and non-linear nature of the human brain makes it challenging to understand its dynamics. As such, insights from theory are needed to help guide computational studies and inform the understanding of brain networks. Here, models of inhibitory networks informed by cortical data were used to explore potential mechanisms leading to increased interneuronal firing and a transition from asynchrony to synchrony that occurred more robustly in hyperexcitable settings. Such synchrony primarily corresponded with fast network oscillations.

However, networks of fast-firing interneurons can also produce slow population output as shown in modeling studies (Ho et al., 2012). The ability of fast-firing inhibitory networks to produce slow population activities was shown to be possible via individual cells having enough of a “kink” in their FI curves that allowed a bistable network mechanism to be present (Ho et al., 2012). The modeled slow population activity (< 5 Hz) is seen *in vitro* using a hippocampal preparation (Wu et al., 2005a,b), and a bistable network mechanism was subsequently leveraged to explain paradoxical changes seen in Rett syndrome mice from the perspective of these same slow population activities (Ho et al., 2014).

A critical difference between the bistable network mechanism of Ho et al. (2012) and bistability related to properties of the ING mechanism (analogous to that presented here) was summarized by Skinner and Chatzikalymniou (2017). In the work of Ho et al. (2012), the mean excitatory drive received by inhibitory cells in the network must be close to their spiking rheobase. The bistability is between states with low or high numbers of fast-firing cells, and this allows slow population activities to come about due to excitatory fluctuations in the system. A similar mechanism could be in play in the work of Schlingloff et al. (2014) where an *in vitro* representation of sharp waves was examined and it was suggested that sharp waves could be generated stochastically from excitatory input. In contrast, for an ING-related bistability, the excitatory drive to the inhibitory cells is not close to spiking rheobase, but as shown by Rich et al. (2016) and in the networks presented here, bistability between synchronized high frequency firing and asynchrony is possible.

There have been numerous studies in the computational literature probing the tendency for networks of inhibitory neurons to synchronize, although these studies typically are done in a more theoretical setting rather than the biologically-motivated manner presented in this study. The interneuron models utilized here exhibit Type I properties in their FI curves [namely, a steep FI curve with an arbitrarily low firing frequency (Hodgkin, 1948)], and neurons with these properties have been a focus of many computational studies of inhibitory synchrony (Chow et al., 1998; Bartos et al., 2002; Brunel and Hansel, 2006; Kopell et al., 2010). As such, the coherent dynamics seen in our inhibitory networks correspond with insights from these more abstract computational studies. This literature contributed to the articulation of the ING mechanism (Traub et al., 1998; Whittington et al., 2000; Tiesinga and Sejnowski, 2009; Wang, 2010) that is most likely driving the coherent dynamics seen in these cortical inhibitory networks. Another seminal study on inhibitory synchrony and ING found that the synchrony promoted by the ING mechanism is most robust when networks are more densely connected and cellular heterogeneity is low (Wang and Buzsáki, 1996), features replicated in the cortically-motivated networks presented here.

In this context we note that computational studies proposing mechanisms for synchronous network oscillations are typically concerned either with purely inhibitory networks (as presented here), purely excitatory networks (Hansel et al., 1995), or networks containing inter- and intra-connected subnetworks of excitatory and inhibitory cells (E-I networks). Crucially, the mechanisms underlying synchrony and their dependence on features, such as cell excitability properties [i.e., the Type I vs. Type II distinction (Hodgkin, 1948)], external drive to the network, and network connectivity can vary significantly depending on the type of network studied. For example, the results of Hansel et al. (1995) imply that an excitatory network made up of cells of the type studied here is highly unlikely to ever synchronize. Similarly, while the Pyramidal Interneuron Network Gamma (PING) mechanism is commonly cited as a mechanism causing synchronous oscillations in E-I networks (Traub et al., 1997; Ermentrout and Kopell, 1998; Whittington et al., 2000; Kopell et al., 2010), recent work has revealed that the predictions of this mechanism are altered by varying individual cellular properties and network connectivity (Rich et al., 2017, 2018).

### 4.3. Limitations and Future Work

The neuron models implemented here used a simplified Izhikevich type integrate-and-fire mathematical structure, informed by a combination of existing literature and in-house experiments. This simplified model has inherent limitations, including the fact that it is a “discontinuous” model that might obscure some physiologically observed network dynamics. With a full repertoire of experimental recordings, one could more fully capture neuronal features and differences, but a consideration of the multiple inhibitory cell types as well as network configurations and properties would also be required to capture the entirety of the biological setting. Indeed, one could consider designing a neuromodulation study using the Blue Brain Project (Markram et al., 2015) to examine this given the insights gleaned from this study; however, this additional complexity has the potential to obscure the underlying mechanisms explaining network activity. This trade-off is a common theme in computational neuroscience, and the desire to uncover a potential mechanism for the neural dynamics of interest motivated the choice of Izhikevich neurons in this study.

The network structure used in this research, a purely inhibitory network, is also simplified from the biology. However, this choice is justified by the focus of this work, which is uncovering a mechanism explaining the dynamics of interneurons in particular prior to seizure initiation, and (as noted in the Introduction) studies of this kind are abundant in the computational literature and have been successful in expanding our understanding of experimentally observed neural behaviors. Because we are investigating the pre-ictal period, we can reasonably assume that no abnormal activity (i.e., synchrony) is present in the pyramidal cells that, in the biological setting, drive inhibitory networks. This justifies our choice of “approximating” this drive with a tonic external input to the purely inhibitory network. These choices facilitate the articulation of our mechanism explaining interneuronal dynamics prior to seizure.

A similar argument as outlined above regarding the use of the Izhikevich model neurons and the purely inhibitory network can also be applied to the synaptic model utilized in this study. One important manifestation of the simplifications inherent in this choice is in the lack of any synaptic delay. Recent work by Tikidji-Hamburyan et al. (2019) has illustrated that synaptic conductance delays may serve an important role in the synchronous dynamics, particularly the clustered dynamics, of purely inhibitory networks. However, we note that the neurons studied here have distinct PRC properties from those of primary focus by Tikidji-Hamburyan et al. (2019) (Type I vs. Type II PRCs in the classical sense), which may explain the lack of any observed two-cluster states in this work. For these reasons we believe that the addition of a synaptic delay would not significantly affect the primary results of this study. Regardless, analyzing the effect of this detail on the networks presented here, and particularly the tendency to exhibit bistability, is a potentially fruitful avenue for future research.

We also note that, while connection probability estimates indicated a value of at least 0.04 was biologically reasonable for these networks (see Methods), simulated networks produced no coherent states with this connection probability. This is perhaps not too surprising given that the cellular models utilized here were only loosely motivated by experiments (see Methods) so that additional estimates of network connectivity are not expected to be precise. However, it is expected that any differences in control and 4-AP models are meaningful since these differences were captured in a comparable fashion (see Methods and Figure 2).

While this mechanism does not describe the entirety of seizure initiation, it does provide a potential avenue by which interneurons in a pathological setting might suddenly synchronize. This is a paramount and necessary “first step” toward an overarching mechanism of a “GABAergic initiation hypothesis.” By showing that this initial step is viable *in silico*, we provide initial justification for further, more biologically detailed study of this hypothesis. With this mechanism in hand, future work can more easily investigate how the dynamics of excitatory cells might affect or interact with this behavior amongst the inhibitory neurons.

For the work here, we focused on differences between control and 4-AP neurons as encapsulated in our models. It is unlikely that utilizing a more realistic noisy synaptic input would affect the primary results of this work, since both noisy (Skinner and Ferguson, 2013) and deterministic (Ferguson et al., 2013) inputs were used in previous hippocampal inhibitory network models without changing insights regarding the transition into synchrony.

While use of a simplified neuron model and network structure enables extensive parameter explorations to be easily done and dynamical aspects, like bistability, to be uncovered, parameter interpretation relative to details of the biological system is less straightforward. However, studies, such as this could help leverage understanding and motivate hypothesis-driven explorations in more detailed models. We note that due to the relatively sparse connectivity of the cortically-motivated inhibitory networks studied, mathematical tools, such as reduction to phase oscillator models as in Hansel et al. (1995) that require an assumption of all-to-all connectivity and weak coupling cannot be easily applied to do further theoretical analyses.

The mechanism proposed in this paper is the first of which the authors are aware that describes the experimentally observed shift in interneuronal activity heralding seizure onset with both biological (Chang M. et al., 2018) and computational (this study) support. This, in turn, provides new and convincing evidence that may help to explain how the hyperexcitability induced by 4-AP causes the cortex to be more vulnerable to seizures, and more generally how interneurons can be involved in the initiation of cortical seizures clinically (Hermes et al., 2017; Honey and Valiante, 2017).

## Data Availability Statement

The code/software described in the paper is freely available online at https://github.com/FKSkinnerLab/CorticalInhibitoryNetwork.

## Author Contributions

SR, MR, KF, FS, and TV contributed to the conception and design of the study. HC performed the electrophysiological experiments. MR and HC performed the necessary research to inform computational parameters with experimental findings. SR wrote and ran the simulations, and wrote the first draft of the manuscript. FS and TV contributed to the writing of the manuscript. All authors contributed to manuscript revision, and read and approved the submitted version.

### Conflict of Interest

The authors declare that the research was conducted in the absence of any commercial or financial relationships that could be construed as a potential conflict of interest.
