# Corrigendum: Inhibitory Network Bistability Explains Increased Interneuronal Activity Prior to Seizure Onset

**DOI:** 10.3389/fncir.2021.727442

**Published:** 2021-09-06

**Authors:** Scott Rich, Homeira Moradi Chameh, Marjan Rafiee, Katie Ferguson, Frances K. Skinner, Taufik A. Valiante

**Affiliations:** ^1^Division of Clinical and Computational Neuroscience, Krembil Research Institute, University Health Network, Toronto, ON, Canada; ^2^Departments of Medicine (Neurology) and Physiology, University of Toronto, Toronto, ON, Canada; ^3^Institute of Biomaterials and Biomedical Engineering, University of Toronto, Toronto, ON, Canada; ^4^Institute of Medical Science, University of Toronto, Toronto, ON, Canada; ^5^Division of Neurosurgery, Department of Surgery, University of Toronto, Toronto, ON, Canada; ^6^Department of Electrical and Computer Engineering, University of Toronto, Toronto, ON, Canada

**Keywords:** epilepsy, seizure, bistability, computational neuroscience, synchrony, inhibitory network, interneurons

In the original article, there was a mistake in Table 1 as published. The values of *d* and *k*_*low*_ were swapped between the Control and 4-AP settings in this table, but correct in the associated code. The corrected Table 1 appears below.

**Table 1:** Parameters used in neuron models.

**Parameter**	**Value (Control)**	**Value (4-AP)**	**Rationale**
*C_*m*_*	73 pF	49 pF	Unpublished in-house experiment
*v_*r*_*	−60.6 mV	−60.6 mV	Ferguson et al. (2013)
*v_*t*_*	−43.1 mV	−43.1 mV	Ferguson et al. (2013)
*v_*peak*_*	2.5 mV	2.5 mV	Ferguson et al. (2013)
*a*	0.01 ms^−1^	0.01 ms^−1^	Parameter influences rheobase and adaptation exhibited by model^*^
*b*	−0.2 nS	−0.4 nS	Parameter influences rheobase and adaptation exhibited by model^*^
*c*	−67 mV	−67 mV	Ferguson et al. (2013)
*d*	0.75 pA	1.25 pA	Parameter influences rheobase and adaptation exhibited by model^*^
*k_*low*_*	0.6 nS/mV	0.4 nS/mV	Parameter influences rheobase and adaptation exhibited by model^*^
*k_*high*_*	2 nS/mV	2 nS/mV	Parameter influences rheobase and adaptation exhibited by model^*^

* *Differences in rheobase and adaptation in control and 4-AP neurons are features shown by Williams and Hablitz (2015) as well as observed in our un-published in-house experiment*.

The authors apologize for this error and state that this does not change the scientific conclusions of the article in any way. The original article has been updated.

## Publisher's Note

All claims expressed in this article are solely those of the authors and do not necessarily represent those of their affiliated organizations, or those of the publisher, the editors and the reviewers. Any product that may be evaluated in this article, or claim that may be made by its manufacturer, is not guaranteed or endorsed by the publisher.
